# A pre-conditioning stress accelerates increases in mouse plasma inflammatory cytokines induced by stress

**DOI:** 10.1186/s12868-015-0169-z

**Published:** 2015-05-07

**Authors:** Yuyan Cheng, Richard S Jope, Eleonore Beurel

**Affiliations:** Department of Psychiatry and Behavioral Sciences and Department of Biochemistry and Molecular Biology, Miller School of Medicine, University of Miami, 1011 NW 15th Street, Gautier Building room 416, Miami, FL 33136 USA

**Keywords:** Cytokines, Depression, Inflammation, Pre-conditioning, Stress, Tumor necrosis factor-α

## Abstract

**Background:**

Major depressive disorder is a prevalent disease that is inadequately treated with currently available interventions. Stress increases susceptibility to depression in patients and rodent models. Depression is also associated with aberrant activation of inflammation, such as increases in circulating levels of interleukin (IL)-1β, IL-6, and tumor necrosis factor-α (TNFα). The two main goals of this study were (i) to identify cytokine changes measuring a broad panel of 19 cytokines, and (ii) to test if a pre-conditioning stress altered the inflammatory response to a subsequent stress.

**Result:**

Stress-induced changes in mouse plasma cytokines were measured by multiplex following administration of one or two daily stresses of inescapable foot shocks using the learned helplessness paradigm for modeling depression-like behavior.

Administration of inescapable foot shocks increased plasma levels of IL-1β, IL-6, TNFα, IL-3, IL-10, IL-13, IL-17A, IL-5, GM-CSF, IL-12(p70), IFN-γ, MIP-1α, MIP-1β, IL-1α, IL-2, KC, RANTES and G-CSF, with peak levels occurring in the range of 6 to 12 hr after stress. Pre-conditioning the mice 24 hr before with an equivalent inescapable foot shock stress resulted in similar magnitudes of increases in most cytokines as occurred after a single stress, but accelerated the increase, causing the levels of most cytokines to peak 1 hr after stress. These results demonstrate that a single stress induces the expression of many cytokines, and that sequential, daily stresses accelerates the rate of cytokine production.

**Conclusions:**

Acute stress broadly activates inflammation in mice, and the inflammatory response is more rapid following repeated stress, actions that may contribute to deleterious effects of stress on depression and other stress-linked diseases.

## Background

Major depressive disorder is a prevalent, progressive, and debilitating disease that afflicts nearly 20% of people in the United States [1]. Current treatments for major depression are inadequate because they take weeks to become effective, and they are often ineffective or not tolerated [2]. New interventions may best be developed by identifying factors that contribute to depression.

There is growing evidence that abnormal inflammatory responses increase susceptibility to depression, and thus may provide new strategies to treat depression [3,4]. This evidence includes consistent findings of increased serum levels in patients with depression of certain pro-inflammatory cytokines, particularly tumor necrosis factor-α (TNFα) and interleukin-6 (IL-6) [5-8]. Moreover, administration of the inflammation stimulant lipopolysaccharide (LPS) or the inflammatory cytokine interferon-α induces depressive symptoms in a significant portion of treated people [9-14]. In addition to clinical evidence, studies in animal models provide further support that inflammation promotes depression-like behaviors. For example, administration of inflammatory cytokines or LPS cause depression-like behaviors in rodents, and these are attenuated by treatment with antidepressants [3,15]. Oppositely, mice with targeted deletion of IL-6 or TNFα receptors display resistance to depression-like behaviors [16-18]. Depression is often induced by psychological stress, and stress induces an inflammatory response [4]. For example, administration of inescapable foot shocks to rodents increased plasma or serum levels of the inflammatory cytokines IL-1β, TNFα, and IL-6 [19-21]. In addition to acute stress, chronic unpredictable mild stress also increased serum levels of TNFα [22]. These studies suggest that the stress-induced inflammatory response may contribute to the pathogenesis of depression. However, a more comprehensive study of cytokine responses to stress would more clearly identify those cytokines that are induced by stress, which is important for identifying the cytokines that may be involved in stress-induced depression.

Substantial evidence has demonstrated that acute or chronic stress sensitizes the inflammatory response to a subsequent immune challenge [23-29]. For example, inescapable tail shock [23] or chronic social stress [27] potentiated the increase of inflammatory cytokines (e.g., IL-1β, TNFα) induced by LPS administration in rodents. However, it is not known if a prior stress influences the inflammatory response to a second stress. Therefore, we examined these topics by comparing the levels of a panel of cytokines in mouse plasma following administration of one or two equivalent stresses on two consecutive days.

## Methods

### Mice

Adult, male C57B/6 mice that were 8–12 weeks old were used for these studies. Mice were housed in groups of 3–5 mice in standard cages in light and temperature controlled rooms and were treated in accordance with NIH and the University of Miami Institutional Animal Care and Use Committee regulations.

### Stress paradigm and plasma collection

Mice were stressed by inescapable foot shocks using a protocol that induces the learned helplessness model of depression-like behavior [30,31]. Mice were placed in one side of a Gemini Avoidance system shuttle box with the gate between the chambers closed. Mice were given 180 foot shocks with an amplitude of 0.3 mA, a 6 sec shock duration, and a randomized inter-shock interval of 5–25 sec. Mouse blood was recovered at decapitation into tubes containing citrate-phosphate-dextrose anticoagulant, and centrifuged at 1,000xg for 10 min, and the plasma was stored in aliquots at −80°C until analyzed. To compare the effects of a single stress and two stresses, some mice were subjected to the identical protocol 24 hr after the first stress.

### Cytokine measurements

A Bio-Plex mouse cytokine multiplex assay was performed according to the manufacturer’s instructions (Bio-Rad, Hercules, CA, USA). Inflammatory molecules measured were IL-1α, IL-1β, IL-2, IL-3, IL-5, IL-6, IL-10, IL-12p40, IL-12p70, IL-13, IL-17A, granulocyte colony-stimulating factor (G-CSF), granulocyte macrophage colony-stimulating factor (GM-CSF), interferon-γ (IFN-γ), keratinocyte chemoattractant (KC), macrophage inflammatory protein (MIP)-1α, MIP-1β, regulated on activation, normal T cell expressed and secreted (RANTES), and TNF-α. Plasma from 3–4 mice was combined, and 50 μl of samples were incubated with antibody-coupled beads. The complexes were washed and then incubated with biotinylated antibody, followed by incubation with streptavidin-PE. Inflammatory molecule levels were determined using a multiplex assay reader (Luminex). The concentrations were calculated using Bio-Plex manager software provided by the manufacturer. Enzyme-linked immunosorbent assays (ELISA) were carried out according to the manufacturer’s instructions (eBioscience).

### Statistical analysis

Statistical significance was analyzed with one-way ANOVA with Bonferroni post-test using Prism software, and p < 0.05 was considered significant.

## Results

### Single stress-induced increases of cytokines in the plasma

**Figure 1:**
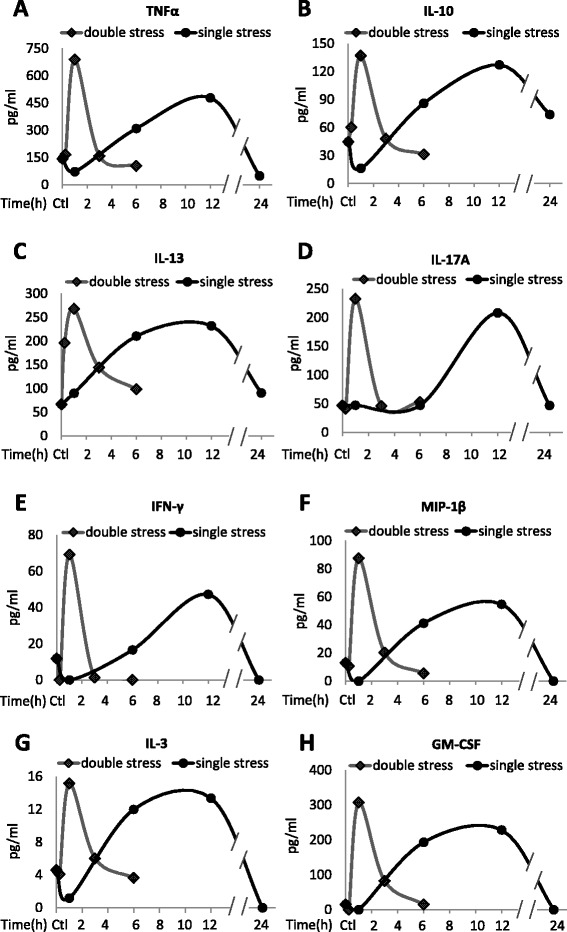
Stress increases plasma cytokines. Plasma **(A)** TNFα, **(B)** IL-10, **(C)** IL-13, **(D)** IL-17A, **(E)** IFN-γ, **(F)** MIP-1β, **(G)** IL-3, and **(H)** GM-CSF levels after inescapable foot shocks. Mice were acutely stressed with inescapable foot shocks once, or two times separated by 24 hr. Plasma was collected 1, 6, 12, and 24 hr after a single session of inescapable foot shocks, and 15 min, 1, 3, and 6 hr after two daily inescapable foot shock sessions. Plasma from three to four mice was pooled and cytokines were measured by multiplex. Values shown as 0 were below the detection limits.

**Figure 2:**
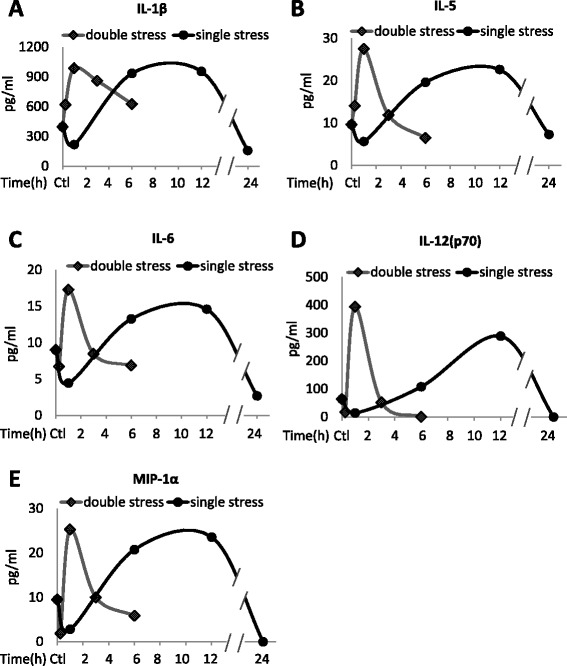
Changes in plasma cytokines after stress. Plasma **(A)** IL-1β, **(B)** IL-5, **(C)** IL-6, **(D)** IL-12(p70), and **(E)** MIP-1α levels after inescapable foot shocks. Mice were acutely stressed with inescapable foot shocks once, or two times separated by 24 hr. Plasma was collected 1, 6, 12, and 24 hr after a single session of inescapable foot shocks, and 15 min, 1, 3, and 6 hr after two daily inescapable foot shock sessions. Plasma from three to four mice was pooled and cytokines were measured by multiplex. Values shown as 0 were below the detection limits.

**Figure 3:**
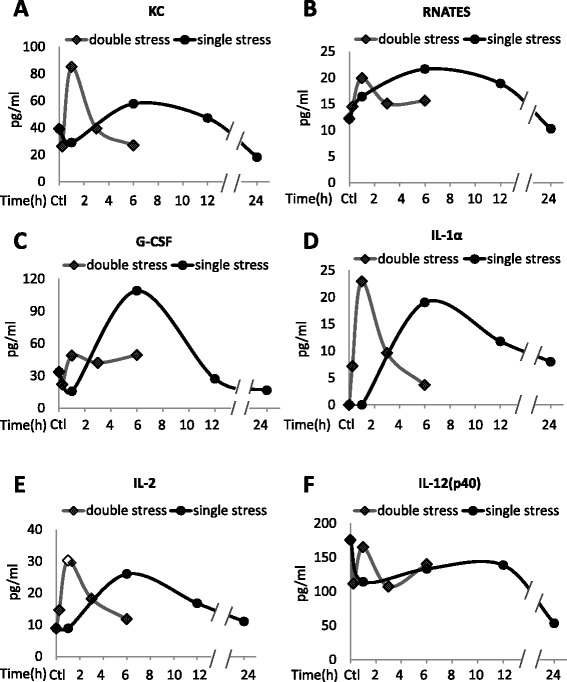
Plasma cytokine levels after stress. Plasma **(A)** KC, **(B)** RANTES, **(C)** G-CSF, **(D)** IL-1α, **(E)** IL-2, and **(F)** IL-12(p40) levels after inescapable foot shocks. Mice were acutely stressed with inescapable foot shocks once, or two times separated by 24 hr. Plasma was collected 1, 6, 12, and 24 hr after a single session of inescapable foot shocks, and 15 min, 1, 3, and 6 hr after two daily inescapable foot shock sessions. Plasma from three to four mice was pooled and cytokines were measured by multiplex. Values shown as 0 were below the detection limits.

**Figure 4:**
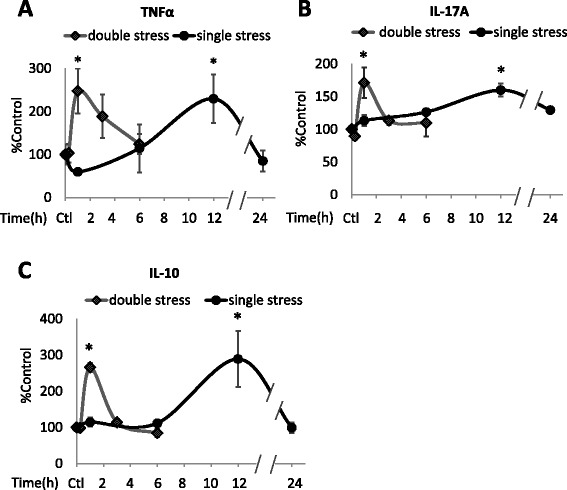
Stress-induced increases in TNFα, IL-17A, and IL-10. Verification of multiplex measurements of **(A)** TNFα, **(B)** IL-17A, and **(C)** IL-10 by ELISA. Plasma levels of TNFα, IL-17A, and IL-10 were measured by ELISA in mice treated with inescapable foot shocks stress once or two times separated by 24 hr. Values are means ± SEM (n = 3–5 mice/group). Data were analyzed by one-way analysis of variance (ANOVA) followed by Bonferroni post-test using Prism software.*p < 0.05 compared to control [Ctl]).

Cytokines in plasma from 3–4 mice were measured by multiplex at 1, 6, 12, and 24 hr after a single stress of inescapable foot shocks. TNFα, IL-10, IL-13, IL-17A, IFN-γ, MIP-1β, IL-3, GM-CSF, IL-1β, IL-5, IL-6, IL-12(p70), and MIP-1α exhibited similar temporal patterns after a single stress (Figures 1 and 2). Among these cytokines, TNFα, IL-10, IL-13, IL-17A, IFN-γ, MIP-1β, and IL-3 increased to a maximum of 3- to 5-fold 12 hr following a single stress, except for a larger increase in GM-CSF, followed by a return to near or below control levels after 24 hr (Figure 1), whereas IL-1β, IL-5, IL-6, IL-12(p70), and MIP-1α levels increased to a maximum of ~1.5- to 3-fold 12 hr after a single stress (Figure 2). Faster increases were exhibited by KC, RANTES, G-CSF IL-1α, and IL-2, which increased to a maximum ~1.5- to 3-fold at 6 hr after a single stress, except for a larger increase in IL-1α, and returned to near control levels after 24 hr (Figure 3). IL-12(p40) levels did not increase, but decreased, after a single stress (Figure 3). ELISAs were used to verify the multiplex results of the plasma levels of TNFα, IL-17A, and IL-10, and both assays showed similar temporal patterns in the induction of the cytokines (Figure 4).

### Stress pretreatment increases the rate of induction of several cytokines induced by a second stress

To test if a prior stress affects stress-induced cytokine production, mice were subjected to identical inescapable foot shocks at a 24 hr interval, a time at which the plasma levels of nearly all cytokines had returned to near basal levels after a single stress. Plasma cytokines were measured at 15 min, 1, 3, and 6 hr after the second stress in 3–4 mice. For most cytokines, a prior stress increased the rate, but not the magnitude, of stress-induced increases in the cytokines (Figures 1, 2 and 3). Most cytokines increased to a maximum of 2- to 6-fold 1 hr after the second stress, demonstrating a more rapid induction than that caused by a single stress, but reaching a similar maximum increase. The rate of return of plasma cytokines to basal levels also was increased, as they reached near control levels 6 hr after the second stress, whereas the return to basal levels was generally slower following a single stress. However, two stresses caused modestly larger increases in the plasma levels of several cytokines, including TNFα (Figure 1a), IFNγ (Figure 1e), MIP-1β (Figure 1f), GM-CSF (Figure 1h), IL-12(p70) (Figure 2d), and KC (Figure 3a), than did a single stress. G-CSF levels increased much less after two stresses than after a single stress (Figure 3c). ELISA measurements of TNFα, IL-17A, and IL-10 confirmed the significant increases 1 hr after two stresses, and the return to basal levels after 6 hr (Figure 4).

## Discussion

This comprehensive evaluation of mouse plasma cytokines revealed substantial increases in the plasma levels of a broad range of cytokines in response to acute stress induced by inescapable foot shocks. The stress-induced increases in plasma cytokines increased rather slowly to reach peak levels after 6 to 12 hr in most cases. However, preconditioning 24 hr previously with acute stress accelerated the stress-induced production of cytokines, whereas preconditioning generally did not alter the amplitude of the increases in cytokine levels. Thus, stress increases the plasma levels of many cytokines and a prior stress increases the rate of the inflammatory response induced by stress but does not change the magnitude of the inflammatory response for most cytokines.

Acute stress induced by a single session of inescapable foot shocks increased the plasma levels of 18 cytokines in mice. Previously, several types of psychological stress have been reported to increase serum levels of IL-1β, IL-6, or TNFα in rodents. Inescapable tail shocks induced an increase of serum IL-1β rapidly after stress, followed by a decrease after 24 hr, but intermediate times between 2 and 24 hr were not evaluated [20,21]. Several studies reported that plasma or serum IL-6 levels increased rapidly after inescapable foot or tail shocks, immobilization, restraint, and exposure to a novel open-field [19,21,32-34]. Serum TNFα levels, but not IL-6 or IL-10, were reported to increase after chronic mild stress [22]. In a more comprehensive evaluation of plasma cytokines following stress, we found that in addition to IL-1β, IL-6 and TNFα, inescapable foot shocks also increased plasma levels of IL-3, IL-10, IL-13, IL-17A, IL-5, GM-CSF, IL-12(p70), IFN-γ, MIP-1α, MIP-1β, IL-1α, IL-2, KC, RANTES and G-CSF. Peak plasma cytokine levels were found to be later than times examined in the previous studies noted above, with maximum levels occurring in the range of 6 to 12 hr. This temporal difference may be due to different stresses that were studied, or to a focus on early times after stress in previous reports. The signaling mechanisms causing increased plasma cytokines following stress have not been clearly established. Stress-induced plasma cytokines have been suggested to originate from the spleen, pituitary gland, and circulating immune cells, such as macrophages [35-40]. Adrenalectomy and adrenergic receptor antagonists attenuated stress-induced IL-1β and IL-6 increases [19,21,35,39], indicating that stress-hormones mediate stress-induced cytokine increases. Regardless of the mechanism, it is evident that acute stress substantially elevates inflammation, which may contribute to reports that stress exacerbates numerous diseases, such as depression, cardiovascular disease, cancer, and aging [4,41-44].

We also tested if a prior stress influences the inflammatory response to stress because previous studies reported that a previous stress increased the inflammatory response to LPS. For example, prior inescapable tail shocks increased LPS-induced plasma levels of TNFα, IL-6, and IL-1β at 1 to 3 hr after LPS treatment [23,24,36]. Also, prior chronic mild stress increased serum TNFα and IL-10 levels 2 hr after LPS treatment [22]. In vitro studies reported that social disruption stress resulted in higher levels of IL-6 and TNFα produced by LPS-stimulated splenocytes [45,46], or splenic CD11b + monocytes [47,48]. We found that exposure to a prior stress of inescapable foot shocks caused a more rapid inflammatory stress-response for most plasma cytokines compared with a single stress. Moreover, we found that a prior stress did not alter the magnitude of stress-induced cytokine levels, except for modestly larger increases of several cytokines. Thus, the major effect of a preconditioning stress was an acceleration of the subsequent stress-induced increase in plasma cytokines. Thus, our data indicates that examination of only one or two times after treatment may not be sufficient to distinguish between stress-potentiated increases in amplitude or changes in the peak times of cytokines that are induced by stress or LPS. The mechanisms by which two stresses induces more rapid cytokine production than a single stress are not known. However, glucocorticoids that are induced by stress have been suggested to play a role in the interactions of stress with LPS-induced cytokines, which may also be relevant to responses to two stressful events. For example, corticosterone pretreatment potentiated LPS-induced levels of TNFα, IL-6, and IL-1β in the liver [49]. Additionally, both pharmacological and surgical suppression of stress-induced glucocorticoids blocked the stress-induced increases of cytokine production after LPS treatment [50]. Therefore it is possible that changes in glucocorticoids contribute to the more rapid release of cytokines after a second stress, which remains to be investigated.

## Conclusion

This study found that in addition to the commonly measured cytokines, IL-1β, IL-6, and TNFα, most cytokines increase in mouse plasma in response to stress. Furthermore, the cytokine inflammatory response to stress is significantly accelerated by prior exposure to stress. This may contribute to the deleterious health effects of repeated stress, and indicate that rapid application of anti-inflammatory interventions may be most appropriate in conditions involving repeated stress.

## Abbreviations

G-CSF: Granulocyte colony-stimulating factor
GM-CSF: Granulocyte macrophage colony-stimulating factor
IFN-γ: Interferon-γ
IL: Interleukin
KC: Keratinocyte chemoattractant
LPS: Lipopolysaccharide
MIP-1α: Macrophage inflammatory protein-1α
MIP-1β: Macrophage inflammatory protein-1β
RANTES: Regulated on activation, normal T cell expressed and secreted
TNFα: Tumor necrosis factor-α

## References

[1] Belmaker RH, Agam G (2008). Major depressive disorder. N Engl J Med.

[2] Nestler EJ, Barrot M, DiLeone RJ, Eisch AJ, Gold SJ, Monteggia LM (2002). Neurobiology of depression. Neuron.

[3] Dantzer R, Kelley KW (2007). Twenty years of research on cytokine-induced sickness behavior. Brain Behav Immun.

[4] Miller AH, Maletic V, Raison CL (2009). Inflammation and its discontents: the role of cytokines in the pathophysiology of major depression. Biol Psychiat.

[5] Dowlati Y, Herrmann N, Swardfager W, Liu H, Sham L, Reim EK (2010). A meta-analysis of cytokines in major depression. Biol Psychiat.

[6] Liu Y, Ho RCM, Mak A (2012). Interleukin (IL)-6, tumour necrosis factor α (TNF-α) and soluble interleukin-2 receptors (sIL-2R) are elevated in patients with major depressive disorder: a meta-analysis and meta-regression. J Affective Disorders.

[7] Pace T, Mletzko T, Alagbe O, Musselman D, Nemeroff C, Miller A (2006). Increased stress-induced inflammatory responses in male patients with major depression and increased early life stress. American J Psychiat.

[8] Steptoe A, Hamer M, Chida Y (2007). The effects of acute psychological stress on circulating inflammatory factors in humans: a review and meta-analysis. Brain Behav Immun.

[9] Dieperink E, Ho SB, Thuras P, Willenbring ML (2003). A prospective study of neuropsychiatric symptoms associated with interferon-alpha-2b and ribavirin therapy for patients with chronic hepatitis C. Psychosomatics.

[10] Hayley S, Poulter MO, Merali Z, Anisman H (2005). The pathogenesis of clinical depression: stressor- and cytokine-induced alterations of neuroplasticity. Neuroscience.

[11] Lotrich FE, Ferrell RE, Rabinovitz M, Pollock BG (2009). Risk for depression during interferon-alpha treatment is affected by the serotonin transporter polymorphism. Biol Psychiatry.

[12] Eisenberger NI, Inagaki TK, Mashal NM, Irwin MR (2004). Inflammation and social experience: an inflammatory challenge induces feelings of social disconnection in addition to depressed mood. Brain Behav Immun.

[13] DellaGioia N, Hannestad J (2010). A critical review of human endotoxin administration as an experimental paradigm of depression. Neurosci Biobehav Rev.

[14] Kullmann JS, Grigoleit JS, Lichte P, Kobbe P, Rosenberger C, Banner C (2013). Neural response to emotional stimuli during experimental human endotoxemia. Hum Brain Mapp.

[15] De La Garza R (2005). Endotoxin- or pro-inflammatory cytokine-induced sickness behavior as an animal model of depression: focus on anhedonia. Neurosci Biobehav Rev.

[16] Chourbaji S, Urani A, Inta I, Sanchis-Segura C, Brandwein C, Zink M (2006). IL-6 knockout mice exhibit resistance to stress-induced development of depression-like behaviors. Neurobiol Dis.

[17] Simen BB, Duman CH, Simen AA, Duman RS (2006). TNFα signaling in depression and anxiety: behavioral consequences of individual receptor targeting. Biol Psychiat.

[18] Koo JW, Duman RS (2008). IL-1β is an essential mediator of the antineurogenic and anhedonic effects of stress. Proc Natl Acad Sci U S A.

[19] Zhou D, Kusnecov AW, Shurin MR, DePaoli M, Rabin BS (1993). Exposure to physical and psychological stressors elevates plasma interleukin 6: relationship to the activation of hypothalamic-pituitary-adrenal axis. Endocrinology.

[20] Nguyen KT, Deak T, Will MJ, Hansen MK, Hunsaker BN, Fleshner M (2000). Timecourse and corticosterone sensitivity of the brain, pituitary, and serum interleukin-1β protein response to acute stress. Brain Res.

[21] Johnson JD, Campisi J, Sharkey CM, Kennedy SL, Nickerson M, Greenwood BN (2005). Catecholamines mediate stress-induced increases in peripheral and central inflammatory cytokines. Neuroscience.

[22] Manikowska K, Mikołajczyk M, Mikołajczak PŁ, Bobkiewicz-Kozłowska T (2014). The influence of mianserin on TNF-α, IL-6 and IL-10 serum levels in rats under chronic mild stress. Pharmacol Rep.

[23] Johnson JD, O’Connor KA, Deak T, Stark M, Watkins LR, Maier SF (2002). Prior stressor exposure sensitizes LPS-induced cytokine production. Brain Behav Immun.

[24] Johnson JD, O’Connor KA, Hansen MK, Watkins LR, Maier SF (2003). Effects of prior stress on LPS-induced cytokine and sickness responses. Am J Physiol Regul Integr Comp Physiol.

[25] Johnson JD, O’Connor KA, Stark M, Watkins LR, Maier SF (2004). The role of IL-1β in stress-induced sensitization of proinflammatory cytokine and corticosterone responses. Neuroscience.

[26] Quan N, Avitsur R, Stark JL, He L, Shah M, Caligiuri M (2011). Social stress increases the susceptibility to endotoxic shock. J Neuroimmunol.

[27] Munhoz CD, Lepsch LB, Kawamoto EM, Malta MB, de Sá LL, Avellar MCW (2006). Chronic unpredictable stress exacerbates lipopolysaccharide-induced activation of nuclear factor-κB in the frontal cortex and hippocampus via glucocorticoid secretion. J Neuroscience.

[28] Frank MG, Baratta MV, Sprunger DB, Watkins LR, Maier SF (2007). Microglia serve as a neuroimmune substrate for stress-induced potentiation of CNS pro-inflammatory cytokine responses. Brain Behav Immun.

[29] Espinosa-Oliva AM, De Pablos RM, Villarán RF, Argüelles S, Venero JL, Machado A (2011). Stress is critical for LPS-induced activation of microglia and damage in the rat hippocampus. Neurobiol Aging.

[30] Polter A, Beurel E, Garner R, Song L, Miller C, Sweatt JD (2010). Deficiency in the inhibitory serine-phosphorylation of glycogen synthase kinase-3 increases sensitivity to mood disturbances. Neuropsychopharmacology.

[31] Beurel E, Harrington LE, Jope RS (2013). Inflammatory T helper 17 cells promote depression-like behavior in mice. Biol Psychiatry.

[32] LeMay LG, Vander AJ, Kluger MJ (1990). The effects of psychological stress on plasma interleukin-6 activity in rats. Physiol Behav.

[33] Kitamura H, Konno A, Morimatsu M, Jung BD, Kimura K, Saito M (1997). Immobilization stress increases hepatic IL-6 expression in mice. Biochem Biophys Res Comm.

[34] Ando T, Rivier J, Yanaihara H, Arimura A (1998). Peripheral corticotropin-releasing factor mediates the elevation of plasma IL-6 by immobilization stress in rats. Am J Physiol.

[35] Jung BD, Kimura K, Kitamura H, Makondo K, Kanehira K, Saito M (2000). Sympathetic activation of hepatic and splenic IL-1beta mRNA expression during oscillation stress in the rat. J Vet Med Sci.

[36] Moraska A, Campisi J, Nguyen KT, Maier SF, Watkins LR, Fleshner M (2002). Elevated IL-1β contributes to antibody suppression produced by stress. J Applied Physiology.

[37] Deak T, Bellamy C, D’Agostino LG (2003). Exposure to forced swim stress does not alter central production of IL-1. Brain Res.

[38] O’Connor KA, Johnson JD, Hansen MK, Wieseler Frank JL, Maksimova E, Watkins LR (2003). Peripheral and central proinflammatory cytokine response to a severe acute stressor. Brain Res.

[39] Blandino P, Barnum CJ, Deak T (2006). The involvement of norepinephrine and microglia in hypothalamic and splenic IL-1β responses to stress. J Neuroimmunology.

[40] Blandino P, Barnum CJ, Solomon LG, Larish Y, Lankow BS, Deak T (2009). Gene expression changes in the hypothalamus provide evidence for regionally-selective changes in IL-1 and microglial markers after acute stress. Brain Behav Immun.

[41] Reiche EMV, Nunes SOV, Morimoto HK (2004). Stress, depression, the immune system, and cancer. Lancet Oncol.

[42] Miller DB, O’Callaghan JP (2005). Aging, stress and the hippocampus. Ageing Res Rev.

[43] Chida Y, Hamer M, Wardle J, Steptoe A (2008). Do stress-related psychosocial factors contribute to cancer incidence and survival?. Nat Clin Pract Oncol.

[44] Dimsdale JE (2008). Psychological stress and cardiovascular disease. J Am Coll Cardiol.

[45] Avitsur R, Padgett DA, Dhabhar FS, Stark JL, Kramer KA, Engler H (2003). Expression of glucocorticoid resistance following social stress requires a second signal. J Leukocyte Biol.

[46] Stark JL, Avitsur R, Hunzeker J, Padgett DA, Sheridan JF (2002). Interleukin-6 and the development of social disruption-induced glucocorticoid resistance. J Neuroimmunology.

[47] Avitsur R, Kavelaars A, Heijnen C, Sheridan JF (2005). Social stress and the regulation of tumor necrosis factor-α secretion. Brain Behav Immun.

[48] Bailey MT, Kinsey SG, Padgett DA, Sheridan JF, Leblebicioglu B (2009). Social stress enhances IL-1β and TNF-α production by Porphyromonas gingivalis lipopolysaccharide-stimulated CD11b + cells. Physiol Behav.

[49] Frank MG, Miguel ZD, Watkins LR, Maier SF (2010). Prior exposure to glucocorticoids sensitizes the neuroinflammatory and peripheral inflammatory responses to E. coli lipopolysaccharide. Brain Behav Immun.

[50] Frank MG, Thompson BM, Watkins LR, Maier SF (2012). Glucocorticoids mediate stress-induced priming of microglial pro-inflammatory responses. Brain Behav Immun.

